# Resolving the slip-rate inconsistency of the northern Dead Sea fault

**DOI:** 10.1126/sciadv.adj8408

**Published:** 2024-03-15

**Authors:** Xing Li, Sigurjón Jónsson, Shaozhuo Liu, Zhangfeng Ma, Nicolás Castro-Perdomo, Simone Cesca, Frédéric Masson, Yann Klinger

**Affiliations:** ^1^ King Abdullah University of Science and Technology (KAUST), Thuwal 23955, Saudi Arabia.; ^2^ Earth Observatory of Singapore, Singapore 639798, Singapore.; ^3^ Department of Earth and Atmospheric Sciences, Indiana University, Bloomington, IN 47405, USA.; ^4^ GeoForschungZentrum (GFZ), 14473 Potsdam, Germany.; ^5^ ITES, EOST Université de Strasbourg, CNRS, 67084 Strasbourg, France.; ^6^ Université Paris Cité, Institut de Physique du Globe de Paris, CNRS, F-75005 Paris, France.

## Abstract

Reported fault slip rates, a key quantity for earthquake hazard and risk analyses, have been inconsistent for the northern Dead Sea fault (DSF). Studies of offset geological and archeological structures suggest a slip rate of 4 to 6 millimeters per year, consistent with the southern DSF, whereas geodetic slip-rate estimates are only 2 to 3 millimeters per year. To resolve this inconsistency and overcome limited access to the northern DSF in Syria, we here use burst-overlap interferometric time-series analysis of satellite radar images to provide an independent slip-rate estimate of ~2.8 millimeters per year. We also show that the high geologic slip rate could, by chance, be inflated by earthquake clustering and suggest that the slip-rate decrease from the southern to northern DSF can be explained by splay faults and diffuse offshore deformation. These results suggest a microplate west of the northern DSF and a lower earthquake hazard for that part of the fault.

## Teaser

The northern Dead Sea fault, just south of the Türkiye earthquakes in 2023, moves only half as fast as previously thought.

## INTRODUCTION

**Fig. 1.**
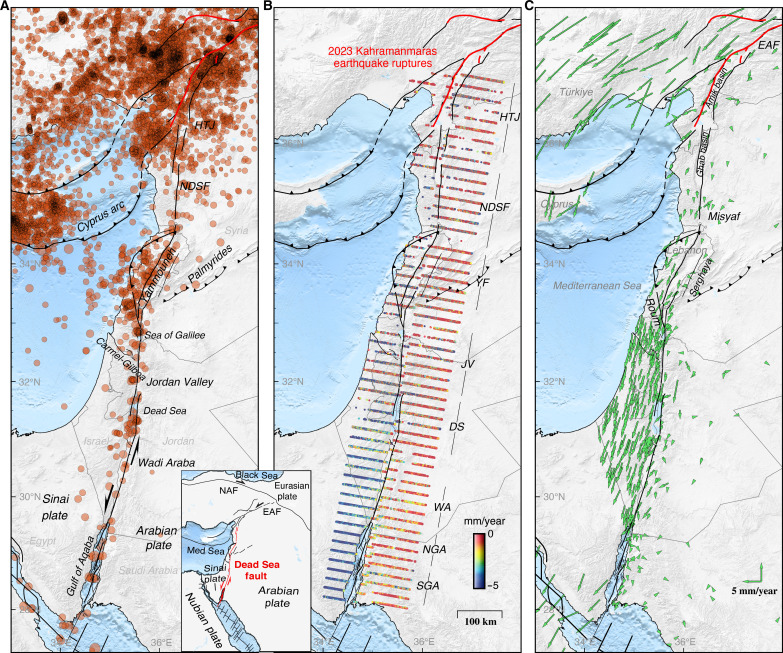
Seismicity and geodetic observations along the DSF. The 1000-km-long Dead Sea left-lateral transform fault system extends from the Red Sea in the south to the Hatay triple junction (HTJ) and East Anatolian Fault (EAF) in the north, here shown with (**A**) earthquake locations (magnitude, >2) from 2000 to 2022 (Euro-Mediterranean Seismological Centre, EMSC), (**B**) fault-parallel velocities with respect to the Arabian plate (negative values indicate southward motion) estimated from time-series analysis of burst-overlap interferometric (BOI) observations, and (**C**) Global Navigation Satellite System (GNSS) velocities from combining multiple sources, again with respect to the Arabian plate. NDSF, northern Dead Sea fault; YF, Yammouneh fault; JV, Jordan Valley; DS, Dead Sea; WA, Wadi Arabah; NGA, northern Gulf of Aqaba; SGA, southern Gulf of Aqaba.

The Dead Sea fault (DSF), the ~1000-km-long left-lateral transform plate boundary between the Sinai and Arabian plates, has been extensively studied since the 1950s (*1*, *2*). The over 2000-year-long historical earthquake catalog of the DSF and numerous geological investigations have made the fault a test bed for a range of hypotheses related to earthquake occurrence patterns and clustering, characteristic earthquakes, fault segmentation, and earthquake interactions (*3*–*7*). Structurally, the DSF can be divided into three main sections from south to north (Fig. 1A). The southern section extends from the Red Sea and the transtensional Gulf of Aqaba along Wadi Arabah and the Jordan Valley, including the extensional step of the Dead Sea basin. The central section consists of the Lebanese restraining bend, which has several splay faults, with the Yammouneh fault as the main through-going structure. Further north, the northern DSF section that runs through the coastal ranges in northwestern Syria consists of the Misyaf fault segment and the faults bounding the Ghab basin and connects to the East Anatolian Fault at the Hatay triple junction in southern Türkiye (*8*), near the southern fault-rupture termination of the first [moment magnitude (*M*_w_) 7.8] mainshock of the 6 February 2023 Kharamanmaraş earthquake doublet (*9*–*11*).

The geodetic and geologic slip-rate estimates are largely consistent for the southern and central DSF. In the south, geodetic slip rates of ~5 mm/year are similar to those from offset geological markers spanning a range of periods since the formation of the DSF (*2*, *12*–*23*). Also, the geodetic slip rate of 3 to 4 mm/year at the Yammouneh fault of the Lebanese restraining bend agrees with the lower bound of the Late Pleistocene/Holocene slip rate of 3.8 to 6.4 mm/year (*24*–*27*). These results, therefore, show no evidence of a secular change in slip rate along the DSF.

Estimated slip rates along the northern DSF from offsets of several archeological sites as well as of geologic and geomorphological features indicate the range of ~4 to 6 mm/year (*28*–*34*), i.e., similar to geologic and geodetic slip rates on the southern DSF. Modeling of GNSS (Global Navigation Satellite System) velocities, on the other hand, indicates that the geodetic slip rate for the northern DSF is only 2.2 to 2.5 mm/year (*35*, *36*), notably lower than estimates of slip rate on other sections of the DSF. Resolving this slip-rate inconsistency on the northern DSF has, however, remained challenging, in part due to the inaccessibility of the fault in Syria during the past decade.

Remote sensing observations, such as from interferometric synthetic aperture radar (InSAR), can often help to overcome inaccessibility challenges. In this case, however, the north-trending strike of the DSF poses a problem, as conventional InSAR inherently has limited sensitivity to north-south displacements (*37*, *38*). While along-track pixel offsets (*39*, *40*) or multiple aperture interferometry (*41*, *42*) provide information about north-south displacements from SAR images, these methods only resolve displacements larger than tens of centimeter and are not capable of resolving millimeter per year interseismic velocities. Burst-overlap interferometry (BOI) from Sentinel-1 radar data, on the other hand, can provide more precise along-track observations, albeit only along narrow swaths where radar bursts overlap (*43*, *44*). Time-series analysis of a large number of BOI data has been shown to allow retrieval of interseismic velocities as low as 5 mm/year (*20*, *45*, *46*); thus, here we extend our work from the Gulf of Aqaba (*20*) and use BOI time-series analysis (see Materials and Methods) for the entire DSF with data from 2014 to 2021 to provide critical constraints on the interseismic deformation and geodetic slip rates along the DSF.

## RESULTS

### Fault-parallel velocities from burst-overlap interferometry

We use 310 ascending and 330 descending radar images from the Sentinel-1A/B satellites, acquired between October 2014 and March 2021, to derive the horizontal velocities along the DSF. The descending along-track velocities closely approximate the fault-parallel motion as most of the DSF is almost parallel to the flight direction of the descending orbit. The horizontal fault-parallel velocities from BOI (i.e., the descending-orbit along-track velocities) attest to subtle left-lateral motion in a northward direction across the DSF (Fig. 1B). In the south, across the Gulf of Aqaba and Wadi Arabah, our BOI velocities (in Fig. 1B) clearly show the ~5 mm/year relative plate motion. Across the Jordan Valley and in Lebanon, the relative motion is also clear in most of the BOI velocities, but the signals are somewhat smaller and noisier than in the south. Further north, across the northern DSF, the fault parallel motion is noisy and less pronounced, due to lower coherence. Similar results are found from time-series analysis of ascending-orbit BOI data (see fig. S1), although the orientation of the ascending-orbit BOI profiles is less favorable to assess the fault-parallel motion.

The northward reduction in fault parallel velocities in the BOI results is in accord with what GNSS observations have shown (*36*). We compiled multiple GNSS results into one solution (*12*, *14*, *15*, *35*, *36*, *47*) and when displayed in the Arabian plate reference frame (Fig. 1C), the relative motion between Arabia and the coastal ranges of Syria is small, clearly smaller than the relative motion across the DSF further south. Both the GNSS and BOI results thus show that the southern Sinai plate (west of the southern DSF) is moving faster to the south (relative to Arabia) than the northern Sinai plate (west of the northern DSF), indicating substantial relative motion between the northern and southern parts of the Sinai plate (*36*) (Fig. 1, B and C).

**Fig. 2.**
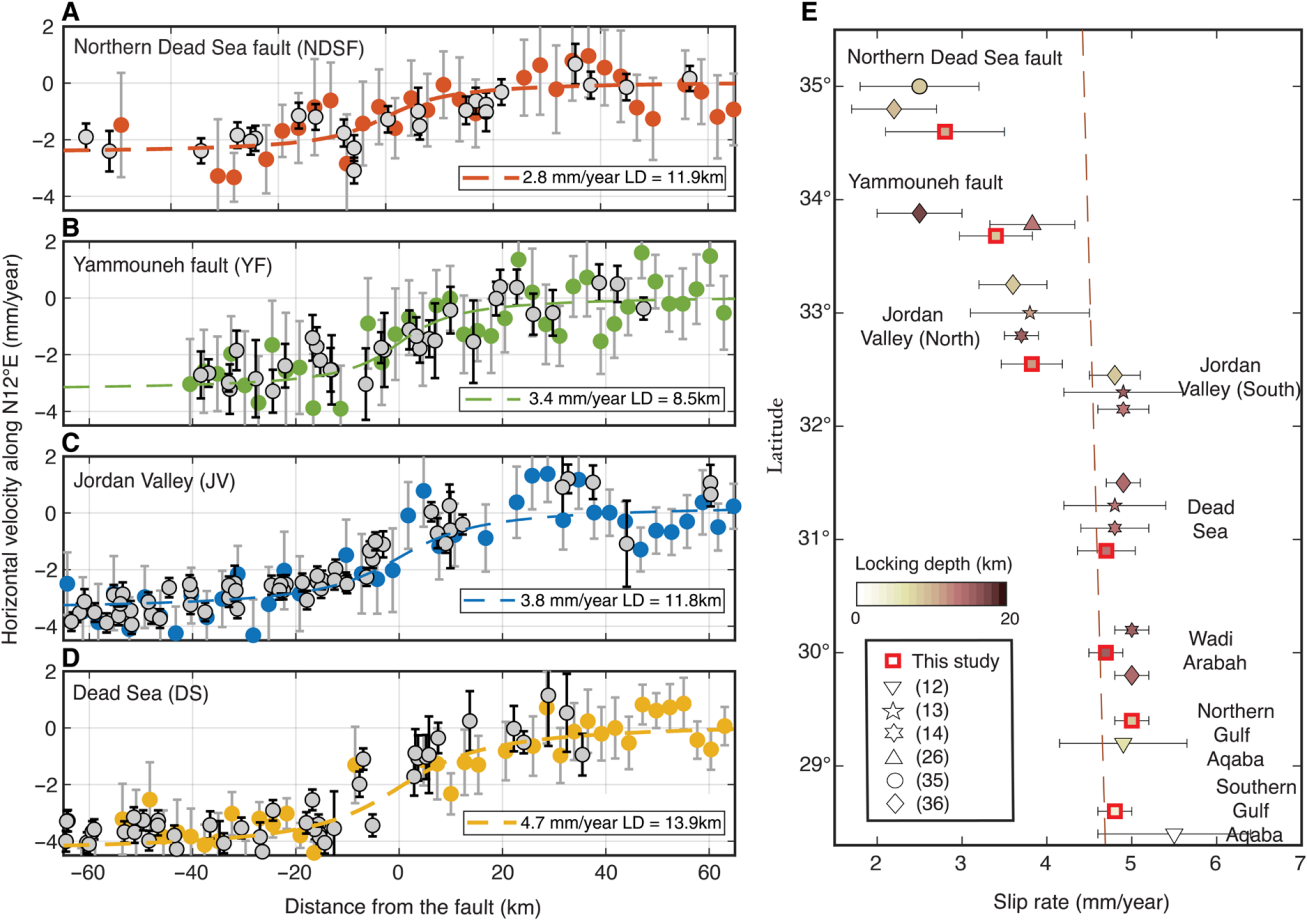
Fault-parallel velocities and estimated geodetic slip rates. (**A** to **D**) Profiles across the DSF at different locations showing the fault-parallel velocity (into direction N12°E) from stacking several BOI profiles (colored circles) in comparison with GNSS velocities (gray); see Fig. 1B for locations and fig. S2 for further profile results. (**E**) Estimated geodetic slip rates and locking depths along the DSF (bold squares, 1-sigma confidence limits; see table S1) compared with other geodetic estimates (other symbols) and the predicted Sinai-Arabia plate motion prediction (*49*) (dashed line). For display purposes, some of the symbols have been slightly shifted vertically from their exact locations of estimation.

To reduce noise and get more robust fault-parallel velocities, we stack all neighboring BOI profiles across each fault segment (as indicated by black lines in Fig. 1B), down-sample the observations and select high-quality points, and assess the uncertainty (see Materials and Methods). The stacked BOI profiles clearly show gradients in the fault-parallel velocities, diagnostic of interseismic strain accumulation, which are consistent with those obtained from GNSS observations (Fig. 2, A to D). Also, the level of noise is comparable between the GNSS and BOI results, but many more BOI observations ensure a more complete mapping of the fault parallel velocities across the different parts of the DSF. As for the BOI and GNSS observations in map view (Fig. 1, B and C), the profiles show the same clear reduction of fault parallel velocities from about 5 mm/year in the south to 2 to 3 mm/year across the northern DSF (Fig. 2, A to D).

For each of the profile data, we estimate the geodetic slip rate (*48*) that confirms the trend of decreasing slip rate from south to north. The best-fitting slip rates for the southern half of the DSF are ~5 mm/year but then decrease to 3.8, 3.4, and 2.8 mm/year for the Jordan Valley, Yammouneh fault, and northern DSF, respectively (Fig. 2E and fig. S2).

The Jordan Valley slip rate decreases from the southern part, just north of the Dead Sea, to the northern part, south of the Sea of Galilee, from 4.3 to 3.5 mm/year (fig. S3). This suggests that a part of the slip rate is transferred from the DSF to the Carmel-Gilboa fault, which has been previously reported in GNSS studies estimating its slip rate in the range of 0.8 to 1.2 mm/year (*14*, *36*). Within the restraining bend, the BOI deformation is not clearly associated with a single fault and we thus presume that the Yammouneh fault accounts for most of the observed deformation. Within uncertainties, the result is consistent with the reported block model rate of 3.8 mm/year (*26*) and with the lower bound of the Late Pleistocene/Holocene slip rate of 3.8 to 6.4 mm/year (*24*).

Together, the descending and ascending BOI results, the GNSS velocities, as well as our modeling results are consistent with the geologic slip rates (*2*, *21*–*23*) for the southern and central parts of the ~1000-km DSF. They are also consistent with the relative plate motion rates according to the latest estimate of the Sinai-Arabia Euler pole (*49*). The exception is the northern DSF, where the geodetic results clearly point to lower slip rates that are inconsistent with the higher estimated geological rates and with what plate motion models predict (Fig. 2E).

## DISCUSSION

### Possible earthquake clustering

Although most estimates of geological slip rates on the northern DSF are in the range of ~4 to 6 mm/year, there is considerable variability in the reported slip rates. A relatively low slip rate of 1.4 to 4.5 mm/year was reported by Searle *et al*. (*34*), a result criticized later by Westaway (*50*) because of inaccurate dating of lava flow units offset by the fault and because of mistaken alignment of piercing points. A faster slip rate of 4.9 to 6.0 mm/year was estimated in the Amik basin based on offset alluvial fans and three archeological sites (*29*, *31*). These rates, however, are not representative of the slip rate on the northern DSF, as the Amik basin is within the Hatay triple junction and thus influenced by the East Anatolian Fault and on-land extensions of faults related to the Cyprus arc. The main geologic slip-rate results of the northern DSF come from the Misyaf segment of the fault, where paleoseismic trenching and archaeoseismic studies of the faulted Al-Harif Roman aqueduct in Syria by four earthquakes during the past 3500 years, each having an estimated offset of ~4 m, yield a slip rate of 4.6 mm/year, or even higher in the range of 4.9 to 6.3 mm/year (*28*, *33*), depending on how the slip rate is calculated.

**Fig. 3.**
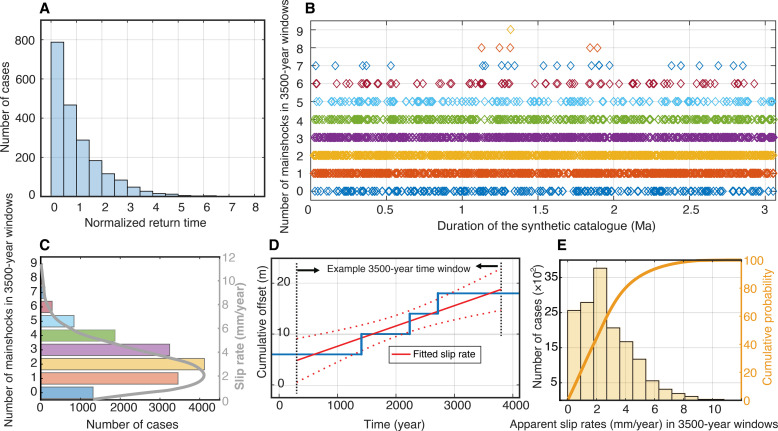
Synthetic earthquake catalog and clustering. Possible earthquake clustering along the northern DSF, assessed from a synthetic earthquake catalog based on the inter-event time statistics observed in the central Dead Sea (*53*). (**A**) Normalized distribution of Dead Sea inter-event times and scaled such that for characteristic 4-m fault-offset mainshocks [as found at Misyaf (*28*, *35*)], it yields a slip rate of 2.8 mm/year (as our geodetic results indicate; Fig. 2A), with an average recurrence interval of ~1430 years. (**B** and **C**) The number of mainshocks is most often one to three in any 3500-year-long time interval of the synthetic catalog, which was generated from randomly drawing 2000 inter-event time samples, yielding ~3-million-year-long synthetic catalog with an accumulated 8-km fault offset. (**D**) Example of estimated apparent slip rate in a 3500-year-long window of the catalog. (**E**) Histogram of ~15,000 apparent slip-rate estimates from sliding the 3500-year window in 200-year-long steps throughout the synthetic catalog. The apparent slip rate is high when, by chance, there are three or more mainshocks in the 3500-year-long window, but low if they are less. In ~12% of the cases, there are four or more mainshocks, resulting in elevated slip rates greater than 4.6 mm/year. Using smaller (3 m) or larger (5 m) characteristic earthquake offsets yield similar results of ~10 and ~14%, respectively (see fig. S4).

We postulate that the high estimated geological slip rate for the Misyaf segment could be elevated due to earthquake clustering during the past 3500 years. Earthquake clustering is widely reported in paleoseismological studies for strike-slip faults where long enough records have been retrieved (*4*, *5*, *51*, *52*). To test this conjecture, we constructed a synthetic earthquake catalog based on the unique 220,000-year-long earthquake record from the central Dead Sea (*53*), which provides a distribution of inter-event times between major earthquakes. Normalizing the inter-event distribution and then scaling it such that characteristic 4-m fault-offset Misyaf mainshocks (*33*) yield a slip rate of 2.8 mm/year, as our geodetic results indicate (Fig. 2A), we compute the probability of fast apparent slip rates within 3500-year time intervals in the synthetic catalog (Fig. 3). About 12% of the time windows include four or more mainshocks, resulting in elevated slip-rates greater than 4.6 mm/year. These results show that it is possible that the estimated 4.6 to 6.3 mm/year fault slip rate on the Misyaf segment of the northern DSF is due to earthquake clustering in the past 3500 years.

There are not many geological slip rate estimates of the northern DSF due to a lack of accurate piercing points and in situ field observations have not been possible for over the past decade. However, one geomorphological study based on a digital elevation model from InSAR yielded a post-Miocene slip rate of 3.3 to 4 mm/year along the northern DSF (*30*), i.e., only slightly higher than the geodetic slip rates. Taking together the geodetic results, the lower bound of this secular slip-rate estimate, and our reasoning above that the high rate on the Misyaf segment could be due to earthquake clustering, the slip-rate inconsistency between geological and geodetic slip rates on the northern DSF has been resolved.

### Tectonic block configuration in the eastern Mediterranean

**Fig. 4.**
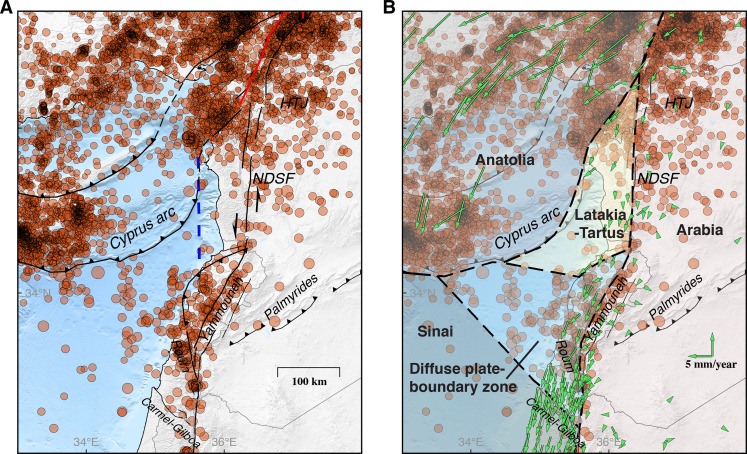
Seismicity and proposed tectonic block configuration in the eastern Mediterranean. (**A**) Seismicity (from EMSC) showing elevated offshore seismicity between Lebanon/Israel and the Cyprus arc. This diffuse seismicity zone appears to bound the Latakia-Tartus microplate to the southwest, with the Cyprus arc bounding it to the west, Hatay triple junction in the north, and the northern DSF in the east. (**B**) Green vectors are GNSS velocities as in Fig. 1C, with respect to stable Arabia, and the blue dashed line in (A) marks the block boundary proposed by Gomez *et al*. (*36*).

The question remains about where the ~2 mm/year difference between the low geodetic slip rate on the northern DSF and the predicted Sinai-Arabia plate motion is accommodated. We explored whether the low geodetic rate could be related to interactions with the offshore Cyprus arc or due to viscoelastic earthquake cycle effects, but neither possibility is likely (see fig. S5). Crustal shortening (1 mm/year) within the Arabian plate over the Palmyrides fold and thrust belt has been suggested (*35*), along with north-south extension (1.5 mm/year) just north of the Lebanese restraining bend. There is, however, scant evidence to support this hypothesis in the GNSS or BOI data (Figs. 1B and 2A). A north-south block boundary just offshore the Syrian coastal ranges has also been proposed in GNSS block modeling (*36*), representing strike-slip motion parallel to the northern DSF (blue dashed line in Fig. 4A). However, results of extensive offshore seismic imaging show no evidence for a strike-slip fault at this location (*54*).

Given the above, it appears that the northern Sinai plate moves separately from the main Sinai plate to the south. The Carmel-Gilboa and Roum faults splay off to the northwest from the DSF and into the Mediterranean, but no clear indication has been found for an offshore plate boundary extending from the coast to the Cyprus arc. Maps of the regional seismicity show distributed seismicity in the area from the coast of northern Israel and Lebanon extending to the Cyprus arc that is markedly stronger than the neighboring offshore seismicity north of Egypt and west of Syria (Fig. 4A). This seismicity suggests a broad zone of diffuse deformation in the eastern Mediterranean, similar to what has been found for some other diffuse oceanic plate boundaries (*55*, *56*). We estimated deviatoric moment tensor solutions for magnitude 3.3 to 5.0 events in this zone and they support (fig. S8 and table S3) that this zone accommodates the relative SSW-NNE extension between the main Sinai plate and its part west of the northern DSF.

Our results thus indicate that the northern Sinai plate is a separate microplate, here named the Latakia-Tartus microplate. It is bounded by the Cyprus arc to the west, the Hatay triple junction to the north, northern DSF to the east, and the Lebanon restraining bend and the offshore diffuse seismicity zone to the south. The GNSS velocities in southern Hatay, southwest of the Amik basin, and in northwestern Syria, clearly show a systematic pattern of velocities different from the velocities to the north (northern Hatay and Anatolia), east (Arabian plate), and south (Fig. 4A). Together, the results resolve the slip-rate inconsistency along the northern DSF and show that the seismic hazard associated with this fault is notably lower than if it were moving at 4 to 6 mm/year. Still, with the last major earthquake on the northern DSF in 1170 (*32*), a slip deficit amounting to about 2.4 m of fault slip has accumulated. While this might appear to indicate that the northern DSF is ripe for another major earthquake, the recent clustering of characteristic 4-m offset events suggests that it might not be the case.

## MATERIALS AND METHODS

We first geometrically aligned the reference and secondary radar images using precise orbits (https://dataspace.copernicus.eu/) for orbital and topographic corrections and formed interferograms (standard InSAR observations) after refining the image co-registration process with the enhanced spectral diversity method (*57*). For the narrow areas of overlapping radar bursts, we subtracted forward- and backward-looking interferograms and generated a redundant network of burst-overlap interferograms with a temporal threshold of 60 days. To reduce ionospheric delays and decorrelation noise, we filtered all the burst-overlap interferograms using a local low-pass filter. In addition, to further reduce noise in the descending-orbit data, we applied a threshold of 0.1 rad (2 cm) in each reconstructed interferogram. We then combined multiple BOI data with a Small Baseline Subset (SBAS) time-series analysis to retrieve the horizontal displacement along the entire DSF. For more details, see the Supplementary Materials.

We inverted for the along-track BOI ground velocity using conventional time-series analysis based on the reconstructed interferograms. However, due to low coherence along the central and northern DSF, the accuracy of each point could not meet the desired level. Therefore, we selected only the high-quality points after velocity stacking. To retain as many coherent observations as possible for velocity stacking, we did not apply any point selection to each overlap velocity. Instead, we applied point selection based on the SD after stacking. The smaller the SD, the better, as the slip rate is assumed to be similar at the same distance from the fault.

To perform the profile stacking, we followed these steps: (i) We down-sampled the observations at 3-km intervals perpendicular to the fault and included all possible overlap areas within each fault segment (as labeled in Fig. 1B). (ii) Using the down-sampled points, we calculated the mean and SD of the points at the same distance from the fault, assuming that the overlap areas are independent of each other. To obtain each median value and estimated 1σ uncertainty (at fault-perpendicular distance *x*), we included all the along-track BOI observations within the same fault-perpendicular distance bin (*x* ±0.6 km). (iii) We applied a threshold to each stacked profile, retaining only the most coherent points.

## Supplementary Materials

### This PDF file includes:

Supplementary Text Figs. S1 to S8 Tables S1 to S3 References
