# Supplementary material for: Resolving the slip-rate inconsistency of the northern Dead Sea fault
Source: Sci Adv. 2024 Mar 15;10(11):eadj8408. doi: 10.1126/sciadv.adj8408 (PMC10942111; doi:10.1126/sciadv.adj8408)
Supplement: Supplementary file 1 — Supplementary Text Figs. S1 to S8 Tables S1 to S3 References [file sciadv.adj8408_sm.pdf]

Science Advances
AAAS

Supplementary Materials for

Resolving the slip-rate inconsistency of the northern Dead Sea fault

Xing Li *et al.*

Corresponding author: Sigurjón Jónsson, sigurjon.jonsson@kaust.edu.sa

Sci. Adv. **10**, eadj8408 (2024)
DOI: 10.1126/sciadv.adj8408

This PDF file includes:

Supplementary Text
Figs. S1 to S8
Tables S1 to S3
References

Supplementary Text

Burst-Overlap Interferometry (BOI)

The TOPS imaging mode of the Sentinel-1 satellites uses azimuth rotation of the antenna beam to achieve complete coverage between successive radar bursts in each sub-swath. The spectral separation between the forward- and backward-looking bursts in areas of overlap between two consecutive bursts can be utilized to obtain an accurate estimate of the azimuthal motion. To begin, we start with a stack of co-registered images, and apply the enhanced spectral diversity method after coarse co-registration with a DEM. The double differential interferogram in the burst-overlap areas can be computed as follows:

$$\Phi_{ovl} = (r_1 \times s_1^*) \times (r_2 \times s_2^*)^* \qquad (1)$$

where r and s represent the reference and secondary images, indices 1 and 2 the forward- and backward-looking views, and the $*$ symbol denotes the conjugate of a complex number. In general, the azimuth displacement Δd_{az} in centimeters can be related to the phase by:

$$\Delta d_{az} = \frac{\Gamma(\Phi_{ovl})}{2\pi\, f_{ovl}\, \tau} r_{az} \qquad (2)$$

where $\Gamma(\cdot)$ is a phase unwrapping operator, f_{ovl} is the spectral separation between the two looks, which is ~4800Hkz for the Sentinel-1 TOPS mode, τ is the azimuth line time, and r_{az} is the azimuth pixel spacing. The double differential interferograms can be subject to notable error sources, including ionospheric delays, orbit errors, and decorrelation noise. Although the split-spectrum technique is commonly used to estimate and remove large-scale ionospheric variations in ALOS-1/2 and Sentinel-1 data (*58-61*), it cannot account for variations within each burst-overlap area. An alternative approach involves modeling the ionospheric delay based on external data, such as TEC maps, but this method also has limitations in achieving high accuracy. To mitigate the effects of strong ionospheric delays, observations suffering from both strong ionospheric delays and decorrelation noise can be removed, but this reduces available observations. Orbit errors can also induce phase ramps in each burst-overlap area for TOPS interferometry due to fast changes in the Doppler centroid inside bursts (*62*). Precise orbits of Sentinel-1 have a 3-D 1-sigma uncertainty of approximately 5 cm (*63*), which corresponds to an azimuth error of 0.002 pixels assuming equivalent uncertainty in 3-D and azimuth pixel spacing of 14 m. Enhanced spectral diversity corrections from double-difference interferograms can be used to estimate azimuth mis-registrations caused by orbit errors with an accuracy of better than 0.001 pixels (*57*) in the image co-registration procedure.

Accuracy of the BOI time-series

The theoretical accuracy for a single burst-overlap interferogram can be written as:

$$\sigma_{ovl} = \frac{1}{2\pi\Delta f_{ovl}\,\tau} \frac{1}{\sqrt{N}} \frac{\sqrt{1-\gamma^2}}{\gamma^2} \qquad (3)$$

where τ is azimuth line time, γ is coherence, N is averaging number, and Δf_{ovl} is the spectral separation of the overlap. The linear system to solve for the velocities V_{BOI} from the BOI phases is then:

$$\phi_{BOI} = GTV_{BOI} \qquad (4)$$

where G is the design matrix and T is time vector in days. Given a full covariance matrix for the enhanced spectral diversity phases C_{BOI}, the covariance matrix of the velocity can be obtained as:

$$C_v = C_{BOI}((GT)^{\mathrm{T}}\, GT)^{-1} \qquad (5)$$

For simplicity, we assume no correlation between interferograms, yielding a diagonal C_{BOI} matrix. The velocity covariances can then be simplified as:

$$C_v = \sigma_{ovl}^2\, ((GT)^{\mathrm{T}}\, GT)^{-1} \qquad (6)$$

The accuracy of the derived BOI velocities is correlated with σ_{ovl}, i.e., the higher the coherence or more averaged observations, the better the accuracy. We compute the time-series accuracy as shown in Fig. S6. In this case, we employ a constant coherence which could overestimate the coherence since long-temporal baseline interferograms could degrade it. For a time-series of 7 years, corresponding to approximately 100 images with a 24-day interval, the accuracy of the BOI velocities can be ~1 cm/yr with medium coherence. The accuracy can be even better (a few millimeters per year) in regions of high coherence, to even one millimeter per year if results from several independent burst-overlap areas are stacked together.

<u>Reconstructing interferograms based on a local low pass filter</u>

Our approach to reducing the influence of ionospheric and decorrelation noise simply involves removing observations with strong ionospheric delays or notable noise and thus reduces the number of observations for the time-series analysis. To address this issue and keep more observations for the estimation, we introduce a more effective method for attenuating noise. After generating the burst-overlap interferograms, we apply a local low-pass filter to reconstruct each interferogram (*64*). The purpose of this filter is to retrieve more observations back and enhance the accuracy of BOI time-series estimation. Specifically, we use a filter with a 20-point window to reduce the high-frequency noise, such as decorrelation noise and tropospheric delays. The linear estimation can be set up as in the following:

$$\begin{bmatrix} 1 & \Delta e_1 & \Delta u_1 \\ 1 & \Delta e_2 & \Delta u_2 \\ \vdots & \vdots & \vdots \\ 1 & \Delta e_n & \Delta u_n \end{bmatrix} \begin{bmatrix} \hat{\phi} \\ \partial M/\partial e \\ \partial M/\partial n \end{bmatrix} = \begin{bmatrix} \phi_1 \\ \phi_2 \\ \vdots \\ \phi_n \end{bmatrix} \qquad (7)$$

where Δe_n Δu_n is the east and north offsets between the target point and surrounding points, ϕ_n are the observations of surrounding points with n = 20, $\hat{\phi}$ is the estimation of the target

point, and $\partial M/\partial e$, $\partial M/\partial n$ are the increment vectors between the target point and the surrounding points. We applied weighted least squares estimation using the coherence as weights to re-estimate the target point.

Elastic dislocation modeling

We fit the elastic dislocation model (*48*) below to each stacked profile result from the descending-orbit data to estimate the fault slip rate s, fault locking depth D, and static offset v_0:

$$v = v_0 + \frac{s}{\pi} tan^{-1}\left(\frac{x}{D}\right) \qquad (8)$$

We used Bayesian inference and implemented the Markov chain Monte Carlo (MCMC) sampler (*65*) to determine the posterior distributions of the model parameters. We set the following model-parameter bounds for the locking depth, slip rate, and offset: 0-30 km, 0-6 mm/year, and -4 mm to 4 mm, respectively. We then used initial 260 random walks to explore the full parameter space, assuming uniform probability distributions, and estimated the maximum a posteriori probability (MAP) solution and corresponding parameter uncertainties from 300,000 MCMC iterations (Fig. S7).

Possible effects from the earthquake cycle and other faults

Interseismic deformation can be influenced by viscoelastic earthquake-cycle effects (*66*), as has been demonstrated in several cases, e.g., along the San Andreas fault and the Kunlun fault (*67-69*). In cases where the earthquake recurrence interval is long or the relaxation time relatively short, the near-fault deformation is notably lower late in the earthquake cycle, than early in the cycle. This could apply for the Dead Sea fault, as most of the fault has not seen major earthquakes for hundreds of years (*32*) and is thus relatively late in the earthquake cycle. Still, the far-field plate motion velocities remain the same and we should see half of the predicted Arabia-Sinai velocity (~2.2 mm/year) at the location of the northern Dead Sea fault (with respect to Arabia), even at times late in the earthquake cycle, if the fault was moving at the expected Arabia-Sinai rate. This is, however, not the case, as the GNSS velocities are notably lower at the northern DSF location (Fig. 1C), indicating viscoelastic earthquake-cycle effects cannot explain the discrepancy between the southern and northern DSF.

Estimated fault-slip rates can be impacted by interseismic deformation at nearby fault systems. The northern DSF is located just south of the Hatay triple junction, with the other two branches of the triple junction being the East Anatolian Fault and the Cyprus arc (Fig. 1A). The Cyprus arc system extends offshore to the southwest from the triple junction, where it is predominantly a left-lateral strike-slip system, and then curves towards the west, where it becomes predominantly a thrust-faulting system south of Cyprus. The estimated left-lateral slip rate on the Cyprus arc system at this location, near the Latakia ridge and west of the northern DSF (assuming Cyprus is on the Anatolian plate), is even larger than the expected 4.6 mm/year Arabia-Sinai velocity. Therefore, given the proximity of this fault system west of the northern DSF, how would interseismic deformation at Latakia ridge affect the deformation (and slip-rate estimates) near the northern DSF?

To model the interaction in between these fault zones, we use the expected relative plate velocities between the Arabian and Sinai plates at the northern DSF (*49*) and the relative motion between Anatolia and Sinai at Latakia ridge (*36*), which yield left-lateral strike-slip rates of 4.4 mm/year and 6 mm/year, and fault-normal (extension) rates of 0.8 mm/year and 2 mm/year (Table S2), at the northern DSF and Latakia ridge, respectively. We then modeled the expected interseismic deformation using a back-slip Okada model and the 1D dislocation model (Equation 8) to explore what influence offshore interseismic deformation would have on the deformation near the northern DSF (Fig. S5). The results show that offshore interseismic deformation would tend to amplify the relative rates across the northern DSF and should thus lead to even higher slip-rate estimates (not lower) than expected from the relative plate motion. Therefore, offshore interseismic motion is not responsible for the low slip rate estimate on the northern DSF. Note that an offshore left-lateral block boundary (fault) like proposed by Gomez et al. (*36*) located just off the Syrian coast, would have a similar influence of increasing the apparent deformation near the northern DSF.

Moment tensor inversion

Full waveform moment tensor inversion has been performed using a Grond, a probabilistic moment tensor inversion algorithm (*70*). We fit 3-components (radial, transversal, vertical) full waveform displacement from broadband seismic stations at regional distances up to 500 km, relying on the following networks: GE-GEOFON (*71*), IS-Israel National Seismic Network, ZR-Desert2000 project, GFZ Potsdam, Germany, Z4-DESIRE (*72*), YB-Continental Dynamics/Central Anatolian Tectonics: Surface to mantle dynamics during collision to escape (CDCAT) (*73*), 3K-TROODOS: Tomography and Receiver function Observations of an Ophiolite using Data Obtained from Seismology (TROODOS) (*74*) and KO-Kandilli Observatory And Earthquake Research Institute (*75*).

Synthetic seismograms are computed using a reflectivity algorithm (*76*) for a regional crustal model (*77*). Observed and synthetic displacements are filtered in different frequency bands (0.05-0.15 Hz, 0.03-0.10 Hz, Hz and 0.02-0.08 Hz), respectively for magnitudes ranging from Mw 3.3 to Mw 5.0. All seismic traces have been visually inspected, and a few traces removed, e.g., in presence of strong noise or gaps. We set up Grond to perform 110,000 iterations. We invert simultaneously the following source parameters: centroid time, centroid location, and moment tensor components, assuming a deviatoric moment tensor, with no isotropic component. The source time function is assumed impulsive. The inversion is simultaneously performed assuming 100 different data weighting configurations. Based on a bootstrap approach we can then estimate mean values and uncertainties for each source parameter (*70*).

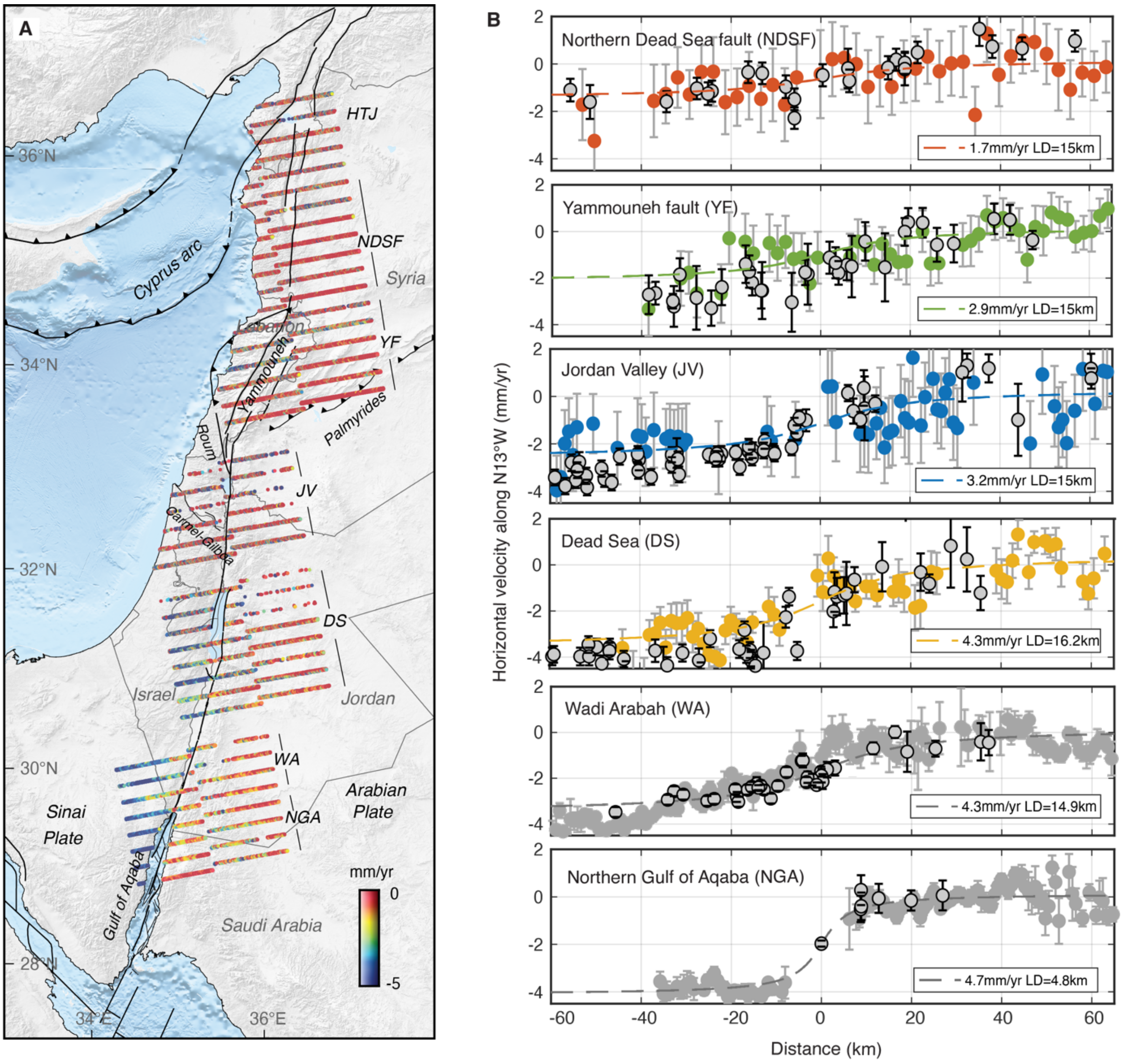

Fig. S1. Horizontal velocities derived from ascending-orbit Burst-Overlap Interferometric analysis, shown with respect to the Arabian plate, in map-view (A) and for across-fault profiles (B). Gray circles denote GNSS velocities for comparison and dashed lines correspond to modeled displacement. The locking depth for the NDSF, YF and JV profiles was fixed to 15 km to avoid unphysical locking depth.

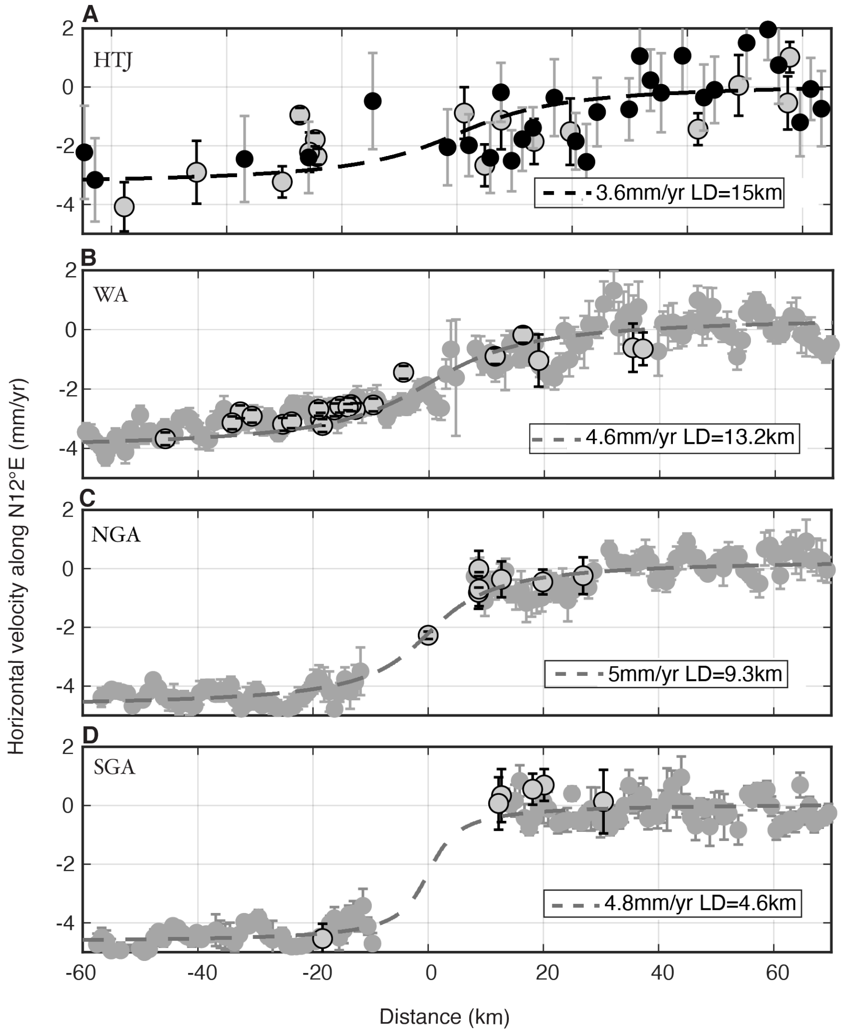

Fig. S2. Four descending-orbit BOI profiles showing fault-parallel velocities (into direction N12°E) in addition to the profiles shown in Fig. 2A. Profiles are derived from stacking of several BOI profiles: (A) near the Hatay triple junction (HTJ), (B) across Wadi Arabah (WA) and (C) northern and (D) southern Gulf of Aqaba (NGA and SGA). Gray circles are fault-parallel GNSS velocities in comparison.

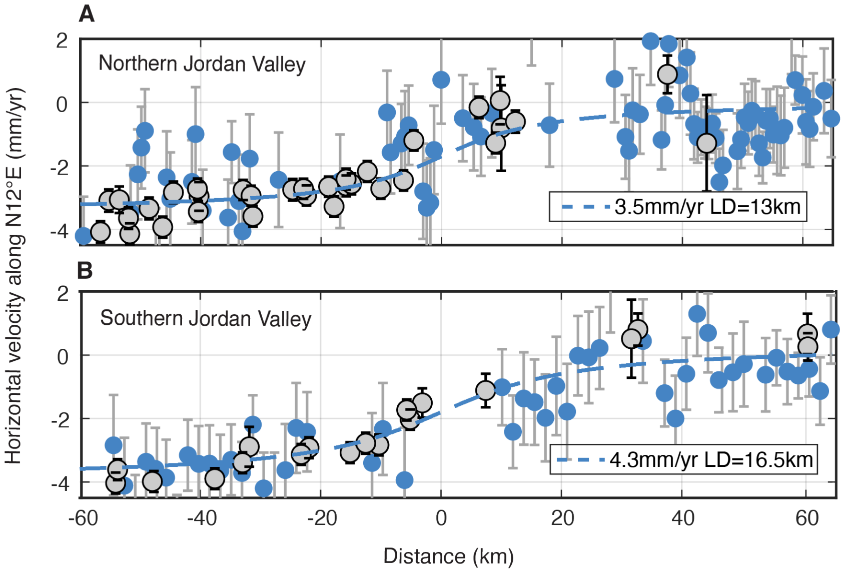

Fig. S3. Two descending-orbit BOI profiles of the fault-parallel velocity (into direction N12°E). Profiles are derived from stacking of several BOI profiles from the northern (A) and southern (B) Jordan valley section of the DSF. Gray circles are fault-parallel GNSS velocities in comparison.

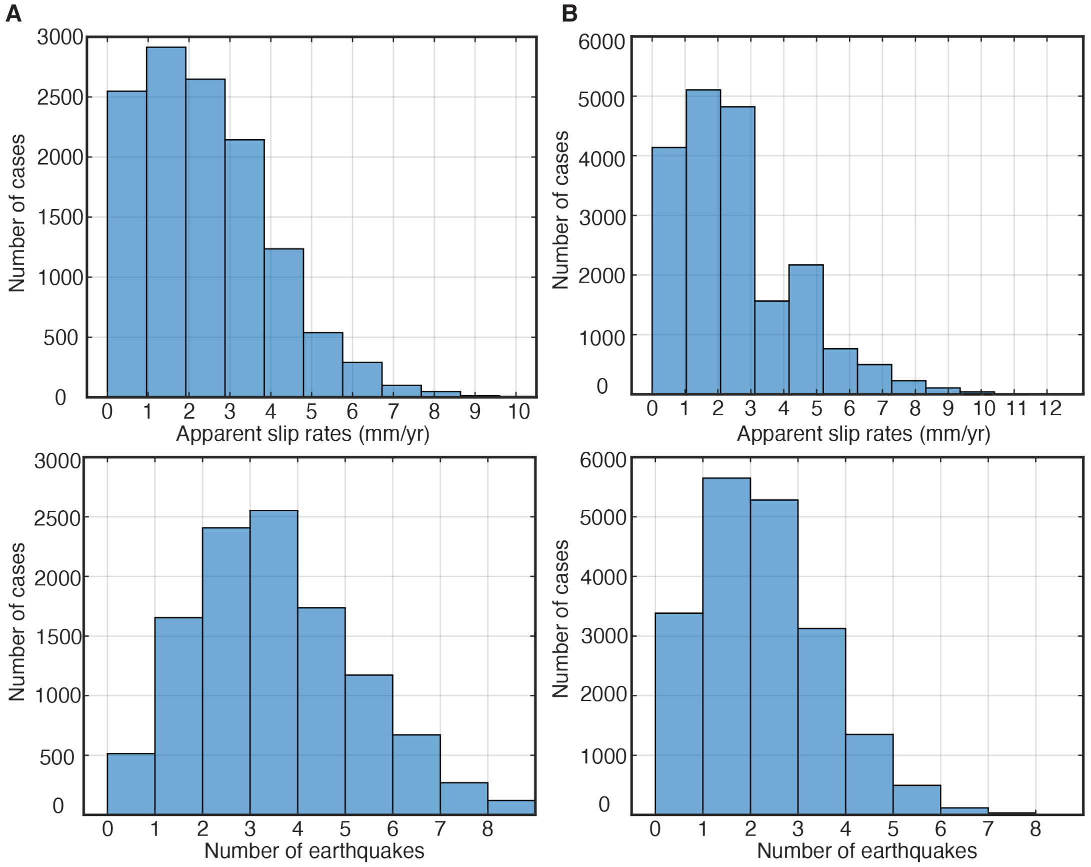

Fig. S4. Estimated slip rates and earthquakes over 3500-year intervals. Histograms of estimated apparent slip rates and number of earthquakes in each 3500-year interval of the simulated earthquake catalogue using a characteristic event size with offsets of (A) 3 m and (B) 5 m.

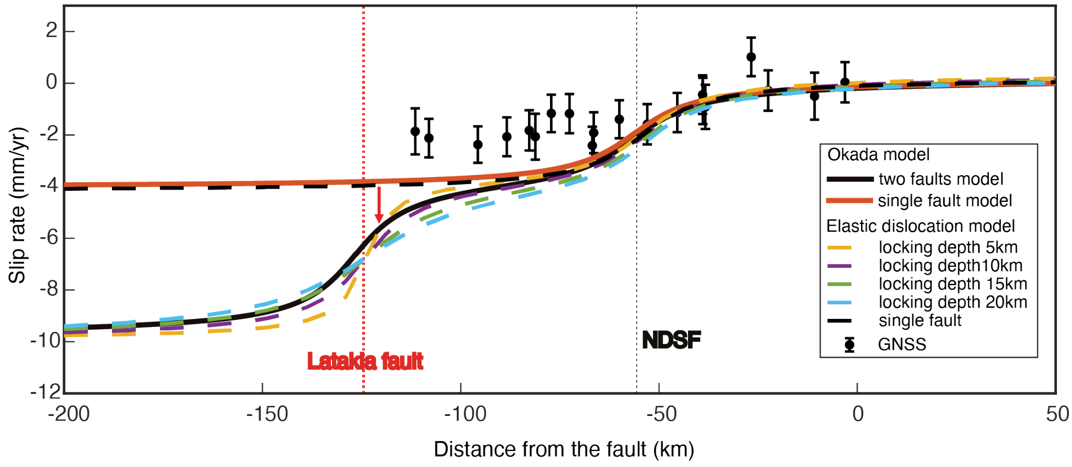

Fig. S5. Fault-parallel velocities with offshore fault. Fault-parallel velocities from GNSS (black dots) across the northern Dead Sea Fault (NDSF) in an Arabia-fixed reference frame (data from Alchalbi et al. (*35*)). For comparison are interseismic model predictions using the Sinai-Arabia plate motion (single fault) from Viltres et al. (*49*), showing that the observed across-fault velocities are too low. By introducing another fault located offshore (two faults), at the Latakia ridge (or similar to what Gomez et al. (*36*) proposed), here denoted as "Latakia fault" with varied locking depth, the combined interseismic deformation of the two faults would make the fit to the data worse.

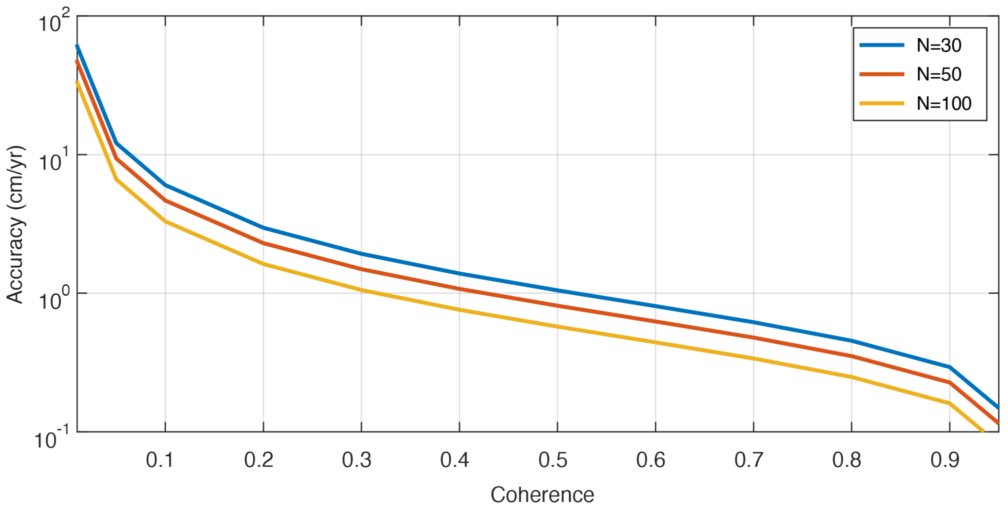

Fig. S6. Time-series accuracy. Estimated time-series accuracy of the BOI estimation as a function of coherence.

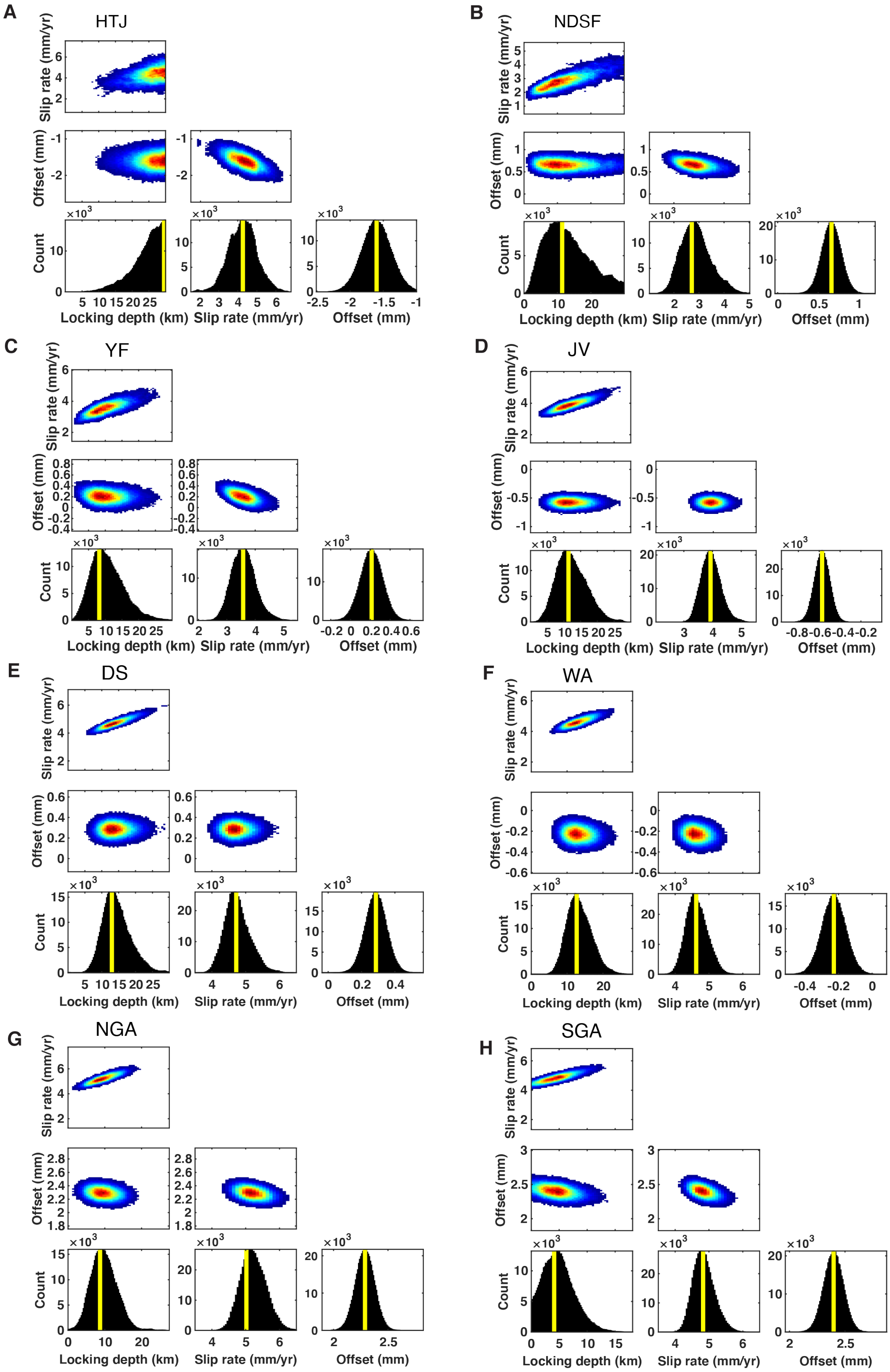

Fig. S7. Estimated 1D and 2D marginal probability distributions of the three model parameters for each of the fault segment (A)-(H) along the DSF, as labeled in Fig. 1B. The yellow line indicates the maximum posterior probability solution from the MCMC analysis.

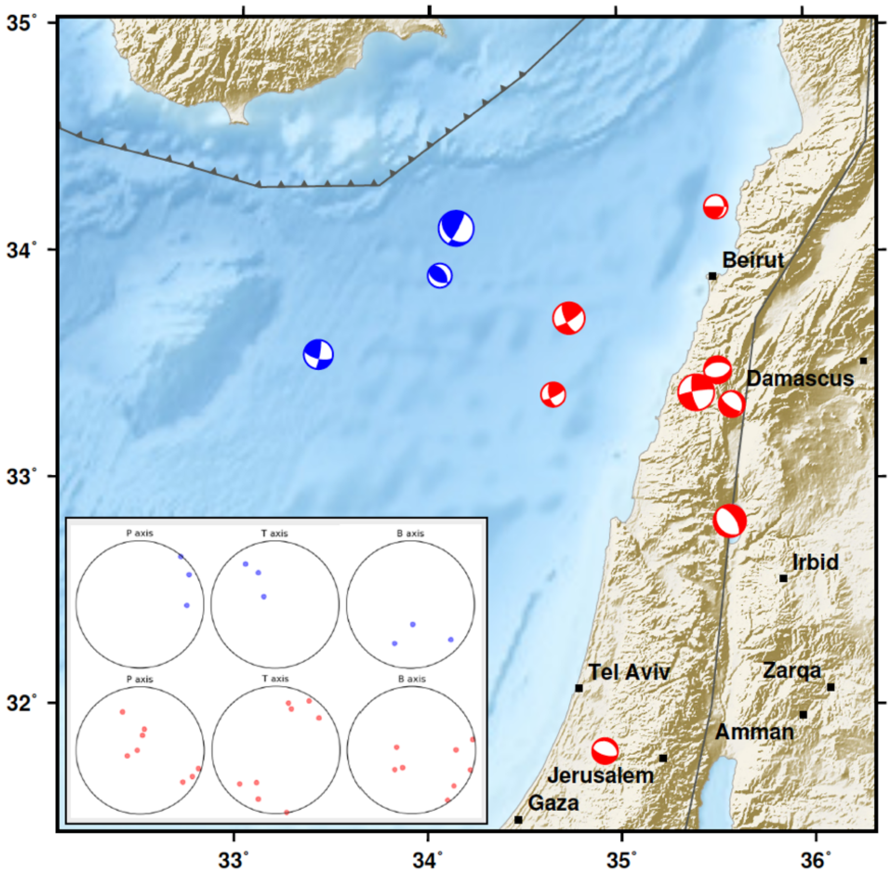

Fig. S8. Estimated deviatoric moment tensor solutions for 11 earthquakes in the eastern Mediterranean. Normal and strike-slip events (in red) show a SSW-NNE oriented T-axis, consistent with SSW-NNE oriented tension. Events close the Cyprus arc (in blue) appear influenced by the subduction zone.

Fault Segment	Dsc(mm/yr)	Locking Depth(km)	Asc(mm/yr)	Locking Depth(km)	GNSS (mm/yr)	Locking Depth(km)	BOI&GNSS (mm/yr)
HTJ	3.6*	/	/	/	3.8*	/	3.8*±0.4
NDSF	2.8±0.6	11.9±6.6	1.7*	/	2.9*	/	2.4*±0.3
YF	3.4±0.4	8.5±5.4	2.9*	/	3.9±0.9	18.7±7.0	3.4*±0.3
JV	3.8±0.3	11.8±4.3	3.2*	/	4.0±0.4	15.2±3.4	3.8*±0.2
DS	4.7±0.3	13.9±4.7	4.3±0.5	16.2±6.4	4.5±0.5	15.9±4.5	4.6±0.3
WA	4.6±0.3	13.2±3.3	4.3±0.4	14.9±5.1	4.6±0.3	18.6±5.7	4.5±0.3
NGA	5.0±0.3	9.3±3.1	4.7±0.3	4.8±2.4	/	/	4.9±0.2
SGA	4.8±0.3	4.6±2.8	/	/	5.1±0.8	1.7±4.3	4.8±0.3

* Locking depth fixed with 15 km

Table S1. Estimations of slip rate and locking depth from geodetic datasets. Slip rate and locking depth estimates from the BOI profile results (Descending and Ascending), GNSS, and combined datasets.

	(°)		(°/Ma)			
Plate pair	Lat	Lon	ω	ω_x	ω_y	ω_z
Anatolian-Sinai	-32.88	218.05	1.3750	-0.9093	-0.7117	-0.7464
Arabia-Sinai	36.34	14.97	0.135	0.1055	0.0282	0.0803

Table S2. Estimated Euler poles used in this study. GNSS-derived Euler poles used in this study for the Anatolia-Sinai and Arabia-Sinai plate pairs from Gomez et al. (*36*) and Viltres et al. (*49*), respectively.

Date	Time	Lat	Lon	Depth	M_W	Strike	Dip	Rake	Strike	Dip	Rake
2007/11/23	22:19:54.0	31.8033	34.9151	12.9	3.6	107.1	64.4	-93.7	295.5	25.9	-82.4
2008/02/15	10:36:21.6	33.3831	35.4076	7.4	5	261.4	83.2	-161.3	169.1	71.5	-7.2
2008/06/12	15:41:02.0	33.3296	35.5948	5.3	3.6	125.1	69.8	-105.3	343.6	25.1	-54.2
2009/04/17	11:03:59.0	33.4813	35.5215	4.1	3.8	76.7	45.9	-95	263.9	44.3	-84.8
2014/05/25	12:22:55.0	34.2009	35.5226	27.7	3.3	271.9	86.4	121.4	7.8	31.6	6.8
2018/07/04	19:45:42.3	32.8136	35.576	4.9	4.5	326.7	62.4	-85.5	137	28	-98.5
2020/11/10	21:49:15.0	33.378	34.6546	28.5	3.4	64.8	75.2	149.5	163.4	60.6	17
2023/02/22	06:01:20.0	33.7152	34.7401	23.9	4.3	58.5	76.4	158.3	153.9	68.9	14.6
2000/12/16	14:27:09.0	33.5532	33.417	28.8	4	103.3	66	8.5	9.8	82.3	155.8
2012/05/11	18:48:32.3	34.1123	34.1431	28	4.9	28.6	81.1	125.1	131	36.1	15.3
2015/07/17	16:41:27.0	33.9041	34.0566	28.4	3.3	113.4	46.1	65.4	326.8	49.1	113.4

Table S3. Estimated deviatoric moment tensor parameters for 11 earthquakes in the eastern Mediterranean and shown in Fig. S8.

REFERENCES

1. L. Dubertret, Problemes de la geologie du Levant. *Bull. Soc. géol. Fr.* **5**, 3–31 (1947).

2. A. M. Quennell, The structural and geomorphic evolution of the Dead Sea rift. *Quart. J. Geol. Soc.* **114**, 1–24 (1958).

3. M. Ferry, M. Meghraoui, N. Abou Karaki, M. Al-Taj, L. Khalil, Episodic behavior of the Jordan Valley section of the Dead Sea fault inferred from a 14-ka-long integrated catalog of large earthquakes. *Bull. Seismol. Soc. Am.* **101**, 39–67 (2011).

4. N. Wechsler, T. K. Rockwell, Y. Klinger, Variable slip-rate and slip-per-event on a plate boundary fault: The Dead Sea fault in northern Israel. *Tectonophysics* **722**, 210–226 (2018).

5. M. Lefevre, Y. Klinger, M. Al-Qaryouti, M. Le Beon, K. Moumani, Slip deficit and temporal clustering along the Dead Sea fault from paleoseismological investigations. *Sci. Rep.* **8**, 4511 (2018).

6. M. Meghraoui, Paleoseismic history of the Dead Sea fault zone. *J. Earthq. Eng.* 1–20 (2015).

7. Y. Klinger, M. Le Béon, M. Al-Qaryouti, 5000 yr of paleoseismicity along the southern Dead Sea fault. *Geophys. J. Int.* **202**, 313–327 (2015).

8. G. Brew, J. Lupa, M. Barazangi, T. Sawaf, A. Al-Imam, T. Zaza, Structure and tectonic development of the Ghab basin and the Dead Sea fault system, Syria, *J. Geol. Soc.* **158**, 665–674 (2001).

9. P. M. Mai, T. Aspiotis, T. A. Aquib, E. V. Cano, D. Castro-Cruz, A. Espindola-Carmona, B. Li, X. Li, J. Liu, R. Matrau, A. Nobile, K. H. Palgunadi, M. Ribot, L. Parisi, C. Suhendi, Y. Tang, B. Yalcin, U. Avşar, Y. Klinger, S. Jónsson, The destructive earthquake doublet of 6 February 2023 in South-Central Türkiye and Northwestern Syria: Initial observations and analyses. *Seism. Rec.* **3**, 105–115 (2023).

10. Z. Jia, Z. Jin, M. Marchandon, T. Ulrich, A.-A. Gabriel, W. Fan, P. Shearer, X. Zou, J. Rekoske, F. Bulut, The complex dynamics of the 2023 Kahramanmaraş, Turkey, M_w 7.8-7.7 earthquake doublet. *Science* **381**, 985–990 (2023).

11. C. Liu, T. Lay, R. Wang, T. Taymaz, Z. Xie, X. Xiong, T. S. Irmak, M. Kahraman, C. Erman, Complex multi-fault rupture and triggering during the 2023 earthquake doublet in southeastern Türkiye. *Nat. Commun.* **14**, 5564 (2023).

12. N. Castro-Perdomo, R. Viltres, F. Masson, Y. Klinger, S. Z. Liu, M. Dhahry, P. Ulrich, J. D. Bernard, R. Matrau, A. Alothman, H. Zahran, R. Reilinger, P. M. Mai, S. Jonsson, Interseismic deformation in the Gulf of Aqaba from GPS measurements. *Geophys. J. Int.* **228**, 477–492 (2022).

13. M. Sadeh, Y. Hamiel, A. Ziv, Y. Bock, P. Fang, S. Wdowinski, Crustal deformation along the Dead Sea Transform and the Carmel Fault inferred from 12 years of GPS measurements. *J. Geophys. Res. Solid Earth* **117**, 2012JB009241 (2012).

14. Y. Hamiel, O. Piatibratova, Spatial variations of slip and creep rates along the Southern and Central Dead Sea fault and the Carmel-Gilboa fault system. *J. Geophys. Res. Solid Earth* **126**, e2020JB021585 (2021).

15. S. McClusky, R. Reilinger, S. Mahmoud, D. Ben Sari, A. Tealeb, GPS constraints on Africa (Nubia) and Arabia plate motions. *Geophys. J. Int.* **155**, 126–138 (2003).

16. S. Wdowinski, Y. Bock, G. Baer, L. Prawirodirdjo, N. Bechor, S. Naaman, R. Knafo, Y. Forrai, Y. Melzer, GPS measurements of current crustal movements along the Dead Sea fault. *J. Geophys. Res. Solid Earth* **109**, 2003JB002640 (2004).

17. M. Le Beon, Y. Klinger, A. Q. Amrat, A. Agnon, L. Dorbath, G. Baer, J. C. Ruegg, O. Charade, O. Mayyas, Slip rate and locking depth from GPS profiles across the southern Dead Sea Transform. *J. Geophys. Res. Solid Earth* **113**, 2007JB005280 (2008).

18. F. Masson, Y. Hamiel, A. Agnon, Y. Klinger, A. Deprez, Variable behavior of the Dead Sea fault along the southern Arava segment from GPS measurements. *C. R. Geosci.* **347**, 161–169 (2015).

19. Y. Klinger, J. P. Avouac, N. Abou Karaki, L. Dorbath, D. Bourles, J. L. Reyss, Slip rate on the Dead Sea transform fault in northern Araba valley (Jordan). *Geophys. J. Int.* **142**, 755–768 (2000).

20. X. Li, S. Jónsson, Y. M. Cao, Interseismic deformation from Sentinel-1 burst-overlap Interferometry: Application to the Southern Dead Sea fault. *Geophys. Res. Lett.* **48**, e2021GL093481 (2021).

21. R. Freund, Z. Garfunkel, I. Zak, M. Goldberg, T. Weissbrod, B. Derin, F. Bender, F. Wellings, R. Girdler, The shear along the Dead Sea rift. *Phil. Trans. Roy. Soc. Lond. A, Math Phys. Sci.* 107–130 (1970).

22. Z. Garfunkel, Internal structure of the Dead Sea Leaky Transform (Rift) in relation to plate kinematics. *Tectonophysics* **80**, 81–108 (1981).

23. S. Joffe, Z. Garfunkel, Plate kinematics of the circum Red Sea—A re-evaluation. *Tectonophysics* **141**, 5–22 (1987).

24. M. Daëron, L. Benedetti, P. Tapponnier, A. Sursock, R. C. Finkel, Constraints on the post∼ 25-ka slip rate of the Yammoûneh fault (Lebanon) using in situ cosmogenic 36Cl dating of offset limestone-clast fans. *Earth Planet. Sci. Lett.* **227**, 105–119 (2004).

25. S. Mahmoud, R. Reilinger, S. McClusky, P. Vernant, A. Tealeb, GPS evidence for northward motion of the Sinai Block: Implications for E. Mediterranean tectonics. *Earth Planet. Sci. Lett.* **238**, 217–224 (2005).

26. F. Gomez, G. Karam, M. Khawlie, S. McClusky, P. Vernant, R. Reilinger, R. Jaafar, C. Tabet, K. Khair, M. Barazangi, Global Positioning System measurements of strain accumulation and slip transfer through the restraining bend along the Dead Sea fault system in Lebanon. *Geophys. J. Int.* **168**, 1021–1028 (2007).

27. F. Gomez, T. Nemer, C. Tabet, M. Khawlie, M. Meghraoui, M. Barazangi, Strain partitioning of active transpression within the Lebanese restraining bend of the Dead Sea fault (Lebanon and SW Syria). *Spec. Publ. Geol. Soc. Lond.* **290**, 285–303 (2007).

28. M. Meghraoui, F. Gomez, R. Sbeinati, J. Van der Woerd, M. Mouty, A. N. Darkal, Y. Radwan, I. Layyous, H. Al Najjar, R. Darawcheh, F. Hijazi, R. Al-Ghazzi, M. Barazangi, Evidence for 830 years of seismic quiescence from palaeoseismology, archaeoseismology and historical seismicity along the Dead Sea fault in Syria. *Earth Planet. Sci. Lett.* **210**, 35–52 (2003).

29. E. Altunel, M. Meghraoui, V. Karabacak, S. H. Akyuz, M. Ferry, C. Yalcmer, M. Munschy, Archaeological sites (tell and road) offset by the Dead Sea fault in the Amik Basin, Southern Turkey, *Geophys. J. Int.* **179**, 1313–1329 (2009).

30. F. Gomez, M. Khawlie, C. Tabet, A. N. Darkal, K. Khair, M. Barazangi, Late Cenozoic uplift along the northern Dead Sea transform in Lebanon and Syria. *Earth Planet. Sci. Lett.* **241**, 913–931 (2006).

31. V. Karabacak, E. Altunel, M. Meghraoui, H. S. Akyuz, Field evidences from northern Dead Sea fault zone (South Turkey): New findings for the initiation age and slip rate. *Tectonophysics* **480**, 172–182 (2010).

32. M. Meghraoui, R. Toussaint, M. Ferry, P. Nguema-Edzang, in *EGU General Assembly Conference Abstracts* (2015), p. 5218.

33. M. R. Sbeinati, M. Meghraoui, G. Suleyman, F. Gomez, P. Grootes, M.-J. Nadeau, H. Al Najjar, Timing of earthquake ruptures at the Al Harif Roman aqueduct (Dead Sea fault, Syria) from archaeoseismology and paleoseismology. *Ancient Earthquakes* **243**, (2010).

34. M. P. Searle, S. L. Chung, C. H. Lo, Geological offsets and age constraints along the northern Dead Sea fault, Syria, *J. Geol. Soc.* **167**, 1001–1008 (2010).

35. A. Alchalbi, M. Daoud, F. Gomez, S. McClusky, R. Reilinger, M. Abu Romeyeh, A. Alsouod, R. Yassminh, B. Ballani, R. Darawcheh, R. Sbeinati, Y. Radwan, R. Al Masri, M. Bayerly, R. Al Ghazzi, M. Barazangi, Crustal deformation in northwestern Arabia from GPS measurements in Syria: Slow slip rate along the northern Dead Sea fault. *Geophys. J. Int.* **180**, 125–135 (2010).

36. F. Gomez, W. J. Cochran, R. Yassminh, R. Jaafar, R. Reilinger, M. Floyd, R. W. King, M. Barazangi, Fragmentation of the Sinai plate indicated by spatial variation in present-day slip rate along the Dead Sea Fault System. *Geophys. J. Int.* **221**, 1913–1940 (2020).

37. J. H. Liu, J. Hu, Z. W. Li, Z. F. Ma, L. X. Wu, W. P. Jiang, G. C. Feng, J. J. Zhu, Complete three-dimensional coseismic displacements due to the 2021 Maduo earthquake in Qinghai Province, China from Sentinel-1 and ALOS-2 SAR images. *Sci. China Earth Sci.* **65**, 687–697 (2022).

38. Y. Fialko, M. Simons, D. Agnew, The complete (3-D) surface displacement field in the epicentral area of the 1999M_W7.1 Hector Mine earthquake, California, from space geodetic observations. *Geophys. Res. Lett.* **28**, 3063–3066 (2001).

39. T. Wang, S. Jónsson, Improved SAR amplitude image offset measurements for deriving three-dimensional coseismic displacements. *IEEE J-Stars* **8**, 3271–3278 (2015).

40. R. Michel, J. P. Avouac, J. Taboury, Measuring ground displacements from SAR amplitude images: Application to the Landers earthquake. *Geophys. Res. Lett.* **26**, 875–878 (1999).

41. H. S. Jung, W. J. Lee, L. Zhang, Theoretical accuracy of along-track displacement measurements from multiple-aperture interferometry (MAI). *Sensors* **14**, 17703–17724 (2014).

42. N. B. Bechor, H. A. Zebker, Measuring two-dimensional movements using a single InSAR pair. *Geophys. Res. Lett.* **33**, 2006GL026883 (2006).

43. H. Jiang, G. Feng, T. Wang, R. Bürgmann, Toward full exploitation of coherent and incoherent information in Sentinel-1 TOPS data for retrieving surface displacement: Application to the 2016 Kumamoto (Japan) earthquake. *Geophys. Res. Lett.* **44**, 1758–1767 (2017).

44. R. Grandin, E. Klein, M. Métois, C. Vigny, Three-dimensional displacement field of the 2015 M_w8.3 Illapel earthquake (Chile) from across- and along-track Sentinel-1 TOPS interferometry. *Geophys. Res. Lett.* **43**, 2552–2561 (2016).

45. Z. F. Ma, S. J. Wei, X. Li, Y. Aoki, J. H. Liu, X. J. Liu, W. F. Mao, S. Yang, N. X. Wang, Q. H. Huang, T. Huang, S. H. Yun, Challenges and prospects to time teries Burst Overlap Interferometry (BOI): Some insights from a new BOI algorithm test over the Chaman fault. *IEEE Trans. Geosci. and Remote Sens.* **60**, 5118219 (2022).

46. M. Lazecký, A. Hooper, P. Piromthong, InSAR-derived horizontal velocities in a global reference frame. *Geophys. Res. Lett.* **50**, e2022GL101173 (2023).

47. R. Reilinger, S. McClusky, P. Vernant, S. Lawrence, S. Ergintav, R. Cakmak, H. Ozener, F. Kadirov, I. Guliev, R. Stepanyan, M. Nadariya, G. Hahubia, S. Mahmoud, K. Sakr, A. ArRajehi, D. Paradissis, A.

Al-Aydrus, M. Prilepin, T. Guseva, E. Evren, A. Dmitrotsa, S. V. Filikov, F. Gomez, R. Al-Ghazzi, G. Karam, GPS constraints on continental deformation in the Africa-Arabia-Eurasia continental collision zone and implications for the dynamics of plate interactions. *J. Geophys. Res. Solid Earth* **111**, 2005JB004051 (2006).

48. J. C. Savage, R. O. Burford, Geodetic determination of relative plate motion in central California. *J. Geophys. Res.* **78**, 832–845 (1973).

49. R. Viltres, S. Jónsson, A. O. Alothman, S. Z. Liu, S. Leroy, F. Masson, C. Doubre, R. Reilinger, Present-day motion of the Arabian plate. *Tectonics* **41**, e2021TC007013 (2022).

50. R. Westaway, Discussion on 'geological offsets and age constraints along the northern Dead Sea fault, Syria'. *J. Geol. Soc. London* **168**, 621–623 (2011).

51. S. Marco, A. Agnon, High-resolution stratigraphy reveals repeated earthquake faulting in the Masada Fault Zone, Dead Sea Transform, *Tectonophysics* **408**, 101–112 (2005).

52. R. Weldon, K. Scharer, T. Fumal, G. Biasi, Wrightwood and the earthquake cycle: What a long recurrence record tells us about how faults work. *GSA Today* **14**, 4–10 (2004).

53. Y. Lu, N. Wetzler, N. Waldmann, A. Agnon, G. P. Biasi, S. Marco, A 220,000-year-long continuous large earthquake record on a slow-slipping plate boundary. *Sci. Adv.* **6**, aba4170 (2020).

54. S. A. Bowman, Regional seismic interpretation of the hydrocarbon prospectivity of offshore Syria. *Geoarabia* **16**, 95–124 (2011).

55. J. A. Wiens, C. S. Crawford, J. R. Gosz, Boundary dynamics: A conceptual-framework for studying landscape ecosystems. *Oikos* **45**, 421–427 (1985).

56. R. G. Gordon, The plate tectonic approximation: Plate nonrigidity, diffuse plate boundaries, and global plate reconstructions. *Annu. Rev. Earth Planet Sci.* **26**, 615–642 (1998).

57. P. Prats-Iraola, R. Scheiber, L. Marotti, S. Wollstadt, A. Reigber, TOPS interferometry with TerraSAR-X. *IEEE Trans. Geosci. Remote Sens.* **50**, 3179–3188 (2012).

58. H. Fattahi, M. Simons, P. Agram, InSAR time-series estimation of the ionospheric phase delay: An extension of the split range-spectrum technique. *IEEE Trans. Geosci. Remote Sens.* **55**, 5984–5996 (2017).

59. C. Liang, E. J. Fielding, Measuring azimuth deformation with L-Band ALOS-2 ScanSAR interferometry. *IEEE Trans. Geosci. Remote Sens.* **55**, 2725–2738 (2017).

60. C. R. Liang, P. Agram, M. Simons, E. J. Fielding, Ionospheric correction of InSAR time series analysis of C-band Sentinel-1 TOPS data. *IEEE Trans. Geosci. Remote Sens.* **57**, 6755–6773 (2019).

61. H. S. Jung, D. T. Lee, Z. Lu, J. S. Won, Ionospheric correction of SAR Interferograms by multiple-aperture interferometry. *IEEE Trans. Geosci. Remote Sens.* **51**, 3191–3199 (2013).

62. A. M. Guarnieri, S. Mancon, S. Tebaldini, in FRINGE Workshop (2015), pp. 1–6.

63. C. Fernández, "Sentinels POD service file format specifications" (Tech. Rep. GMESGSEG-EOPG-FS-10-0075, European Space Agency, 2011).

64. J. H. Liu, J. Hu, Z. W. Li, J. J. Zhu, Q. Sun, J. Gan, A method for measuring 3-D surface deformations with InSAR based on strain model and variance component estimation. *IEEE Trans. Geosci. Remote Sens.* **56**, 239–250 (2018).

65. K. Mosegaard, A. Tarantola, Monte Carlo sampling of solutions to inverse problems. *J. Geophys. Res. Solid Earth* **100**, 12431–12447 (1995).

66. J. Savage, W. Prescott, Asthenosphere readjustment and the earthquake cycle. *J. Geophys. Res.* **83**, 3369–3376 (1978).

67. F. Q. Diao, X. Xiong, R. J. Wang, T. R. Walter, Y. B. Wang, K. Wang, Slip rate variation along the Kunlun Fault (Tibet): Results from new GPS observations and a viscoelastic earthquake-cycle deformation model. *Geophys. Res. Lett.* **46**, 2524–2533 (2019).

68. J. S. Tong, T. Ma, K. R. Shen, H. Y. Zhang, S. P. Wu, A criterion of asphalt pavement rutting based on the thermal-visco-elastic-plastic model. *Int. J. Pavement Eng.* **23**, 1134–1144 (2022).

69. K. L. Wang, Y. J. Zhu, E. Nissen, Z. K. Shen, On the relevance of geodetic deformation rates to earthquake potential. *Geophys. Res. Lett.* **48**, e2021GL093231 (2021).

70. S. Heimann, M. Isken, D. Kühn, H. Sudhaus, A. Steinberg, S. Daout, S. Cesca, H. Bathke, T. Dahm, Grond: A probabilistic earthquake source inversion framework (2018).

71. GEOFON Data Centre, "GEOFON seismic network" [dataset] (Deutsches GeoForschungsZentrum GFZ, 1993).

72. G. Asch, A. Mohsen, B. Braeuer, R. Hofstetter, D. Jaser, R. El-Kelani, M. Weber, "DESIRE - Seismology (2006/2008)" [dataset] (GFZ Data Services, 2006).

73. E. Sandvol, "Continental dynamics/central anatolian tectonics: Surface to mantle dynamics during collision to escape" [dataset] (International Federation of Digital Seismograph Networks, 2013).

74. I. Bastow, C. Ogden, S. Pilidou, I. Dimitriados, P. Iosif, C. "Constantinou, TROODOS: Tomography and receiver function observations of an Ophiolite using data obtained from seismology" (International Federation of Digital Seismograph Networks, (2016).

75. "KO: Kandilli Observatory And Earthquake Research Institute (KOERI)" [dataset] (International Federation of Digital Seismograph Networks, 1971).

76. R. J. Wang, A simple orthonormalization method for stable and efficient computation of Green's functions. *Bull. Seismol. Soc. Am.* **89**, 733–741 (1999).

77. C. Bassin, The current limits of resolution for surface wave tomography in North America. *Eos. Trans. AGU* **81**, F897 (2000).
